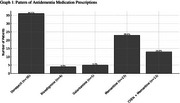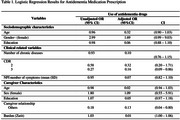# Patterns in the use of antidementia drugs among people with dementia in Brazil

**DOI:** 10.1002/alz70858_104050

**Published:** 2025-12-26

**Authors:** Fernanda M. de Faria, Carolina Godoy, Lucas Martins Teixeir, Laiss Bertola, Ari Alex Ramos, Fabiana A F da Mata, Haliton Alves de Oliveira Júnior, Cleusa P Ferri

**Affiliations:** ^1^ Federal University of São Paulo, São Paulo, Sao Paulo, Brazil; ^2^ Universidade Federal de São Paulo, São Paulo, São Paulo, Brazil; ^3^ Universidade Federal de São Paulo (UNIFESP), São Paulo, São Paulo/SP, Brazil; ^4^ University of Sao Paulo Medical School, Sao Paulo, Sao Paulo, Brazil; ^5^ Federal University of São Carlos, São Carlos, São Paulo, Brazil; ^6^ Hospital Alemão Oswaldo Cruz, São Paulo, São Paulo, Brazil; ^7^ Federal University of Sao Paulo (UNIFESP), Sao Paulo, Brazil

## Abstract

**Background:**

A total of 140 individuals diagnosed with dementia, and their 140 primary caregivers were interviewed. Participants were categorized in two groups: those using antidementia medications (cholinesterase inhibitors and memantine) and those who were not using them.

**Methods:**

Data collection was conducted through structured interviews using questionnaires about disease progression, health services use, changes in mood and behavior, quality of life, and caregiving needs for both patients and caregivers. Additionally, other scales were applied: (CDR), (NPI‐Q), (JHDCNA 2.0), and DEMQOL. We used logistic regression to identify sociodemographic and clinical characteristics associated to the use of antidementia medication.

**Results:**

Among the 140 older adults with dementia, 57.8% (*n* = 81) were receiving pharmacological treatment, including donepezil (44.4%, *n* = 36), rivastigmine (4.9%, *n* = 4), galantamine (6.2%, *n* = 5), memantine (28.4%, *n* = 23), and cholinesterase inhibitors (ChEIs) in combination with memantine (16%, *n* = 13). The mean age was significantly lower in the group using dementia medications (79.92 ± 8.0 years) than in those not using such medications (83.25 ± 7.4 years, *p* = 0.013). Dementia severity, assessed by the Clinical Dementia Rating (CDR), differed between groups (*p* = 0.029), with a higher prevalence of severe cases in the group not receiving medications (52.5% vs. 30.8%). In the logistic regression analysis, patient age was not significantly associated with medication use in the adjusted model (OR = 0.32). Patients with more severe dementia (CDR 3) were more likely **not** to use medications (adjusted OR = 0.27; 95% CI: 0.09–0.86), indicating a significant inverse association with disease severity. Patients with non‐spousal caregivers had a lower likelihood of using medications (adjusted OR = 0.18; 95% CI: 0.04–0.80). By contrast, greater caregiver burden, as measured by the Zarit scale, was positively associated with medication use (adjusted OR = 1.03; 95% CI: 1.00–1.06).

**Conclusion:**

The study demonstrates that older adults in lower age brackets and with milder dementia were more likely to use pharmacological treatments, whereas those cared for by non‐spousal caregivers exhibited lower medication use. Furthermore, caregiver burden was positively correlated with the likelihood of using cholinesterase inhibitors (ChEIs), either alone or in combination with memantine.